# Cytogenetics and FLT3-ITD mutation predict clinical outcomes in non transplant patients with acute myeloid leukemia

**DOI:** 10.1186/s40164-019-0127-z

**Published:** 2019-01-30

**Authors:** Pimjai Niparuck, Nittaya Limsuwanachot, Sulada Pukiat, Pichika Chantrathammachart, Budsaba Rerkamnuaychoke, Sutada Magmuang, Sithakom Phusanti, Kochawan Boonyawat, Teeraya Puavilai, Pantep Angchaisuksiri, Artit Ungkanont, Suporn Chuncharunee

**Affiliations:** 1 Division of Hematology, Department of Medicine, Ramathibodi Hospital, Mahidol University, Bangkok, Thailand; 2 Human Genetics Laboratory, Department of Pathology, Ramathibodi Hospital, Mahidol University, Bangkok, Thailand; 3 Department of Medicine, Chakri Naruebodindra Medical Institute, Mahidol University, Bangkok, Thailand

**Keywords:** Acute myeloid leukemia, Cytogenetics, FLT3-ITD, NPM1, CEBPA

## Abstract

**Background:**

Cytogenetic abnormalities and mutated genes indicate the role of consolidation therapy with hematopoietic stem cell transplantation (HSCT) or chemotherapy in acute myeloid leukemia (AML). In this study, we conducted a retrospective study in adult AML patients with newly diagnosed with de novo AML who did not undergo HSCT, to study long term relapse free survival (RFS) and overall survival (OS) after consolidation chemotherapy.

**Methods:**

We recruited 141 consecutive AML patients during January 2010–June 2017, the patients received induction chemotherapy with standard dose Ara-C and Idarubicin (7 + 3 or 5 + 2 regimen) followed by intermediate (IDAC) or high dose Ara-c (HiDAC) consolidation therapy.

**Results:**

Normal karyotype, complex, favorable, intermediate and adverse chromosomal aberrations were found in 59%, 16%, 5%, 14% and 6%, respectively. Mutated NPM1, FLT3-ITD and CEBPA genes in CN-AML were seen in 33%, 18% and 19%, respectively. A 5 year follow up, 5y-RFS was 16% and 5y-OS was 14% in the whole study population. 5y-RFS and 5y-OS in patients completed 4 cycles of consolidation therapy were 25% and 40%, respectively. Adverse cytogenetic risk and mutated FLT3-ITD were significantly associated with poor RFS (9 and 15 months, respectively) and OS (14 and 16 months, respectively), whereas patients with mutant NPM1 had favorable outcomes (RFS/OS = 51/63 months). Patients receiving 4 cycles of consolidation therapy had significantly impacts on median RFS and OS compared with those treated with 1 or 2 cycles; 15 versus 11 months (*p* = 0.006) and 31 versus 15 months (*p* < 0.001), respectively.

**Conclusions:**

Cytogenetic and mutation tests for FLT3-ITD, NPM1 and CEBPA genes were meaningful for predicting outcomes in adult AML patients. Adverse cytogenetic abnormalities and FLT3-ITD mutation showed dismal RFS and OS.

## Background

Acute myeloid leukemia (AML) is the heterogeneous disease which caused by various molecular and cytogenetic abnormalities driven leukemogenesis, including chromosome abnormalities, gene mutations, RNA splicing, cohesin complexity and epigenetic alterations. Currently, there are a number of gene alterations associated with treatment outcomes in AML patients that performed by next generation sequencing technique, however, this method is not yet implemented in many medical centers. Based on work by Schlenk et al. [1], the data has been shown that mutations of FMS related tyrosine kinase 3 (FLT3)-internal tandem duplications (ITDs), nucleophosmin1 (NPM1) and CCAAT/enhancer binding protein α gene (CEBPA) can indicate the prognosis of disease in normal karyotype AML. According to 2017 European LeukemiaNet (ELN) recommendations, mutant NPM1 gene with wild type FLT3-ITD or low allele burden of FLT3-ITD mutation and bi-allelic CEBPA mutation are considered to be the favorable prognostic risk stratification for AML, whereas high allele burden of FLT3-ITD mutation with or without mutant NPM1 is classified as intermediate and unfavorable risk, respectively. Other gene mutations associated with adverse outcomes for AML are mutated RUNX1, ASXL1 and TP53. AML patients with t(8;21)(q22;q22.1), inv(16)(p13.1q22), t(16;16)(p13.1;q22), mutated NPM1 with wild type FLT3-ITD or low FLT3-ITD allele burden or biallelic CEBPA gene mutation are recommended to receive consolidation therapy with 2–4 cycles of intermediate dose Ara-C (IDAC), whereas allogeneic hematopoietic stem cell transplantation (Allo-HSCT) is preferred for consolidation treatment in AML without favorable cytogenetics, AML with adverse risk gene mutations or AML with wild-type NPM1 and CEBPA genes [2]. Currently, the FLT3 inhibitor combined with chemotherapy or hypomethylation agent improves the treatment outcomes in AML patients with FLT3 mutation [3–5]. However, there were some differences between mutation patterns of AML among Asian and Western populations; the mutations of NPM1, FLT3-ITD, FLT3-tyrosine kinase domain (TKD) and CEBPA were found in 16% (Asian) versus 30% (Western), 11% (Asian) versus 23% (Western), 9% (Asian) versus 10% (Western) and 21% (Asian) versus 9% (Western), respectively [6]. We therefore performed a retrospective study to analyze long term relapse free survival (RFS) and overall survival (OS) in adult non-transplant patients with de novo AML receiving IDAC or high dose Ara-C (HiDAC) based on clinical data, cytogenetic patterns and mutations of NPM1, FLT3-ITD and CEBPA.

## Materials and methods

### Patients

The study was conducted during 1 January 2010–30 June 2017, we enrolled 141 consecutive patients with newly diagnosed with de novo AML who did not undergo allo-HSCT. All patients were older than 15 years old and had the results of cytogenetic, FLT3-ITD, NPM1 and CEBPA gene mutation analysis. The patients receiving supportive and hypomethylating agent therapies were allowed to enroll into the study. The patients who received prior chemotherapy or previous allogeneic or autologous HSCT were excluded. The patients with acute promyelocytic leukemia, secondary AML or therapy related AML were not eligible for the study.

### Chemotherapy protocols

#### Induction chemotherapy

The patients younger than 60 years of age received induction chemotherapy with intravenous (i.v.) Ara-C 100 mg/m^2^/day for 7 consecutive days together with i.v. idarubicin 12 mg/m^2^/day for 3 consecutive days (7 + 3 regimen). The patient aged ≥ 60 years or < 60 years with creatinine clearance < 50 mL/min or had septicemia or pulmonary infection at diagnosis of AML were treated with 5 + 2 regimen that consisted of 5 days of i.v. Ara-C 100 mg/m^2^/day (d1–5) combined with 2 days of i.v. idarubicin 12 mg/m^2^/day (d1–2). The bone marrow (BM) study was re-evaluated 28 days after induction therapy. The patient aged ≥ 70 years received azacitidine 100 mg/day subcutaneously for 7 consecutive days, every 4 weeks, and the disease response was re-evaluated after 4–6 cycles. Complete remission (CR) was defined as BM blasts < 5%, absolute neutrophil count ≥ 1000/μL, platelet count ≥ 100,000/μL, absence of circulating blasts and absence of extramedullary disease.

#### Consolidation chemotherapy

Patients aged < 60 years achieving CR after induction chemotherapy were treated with the first cycle of consolidation chemotherapy with the same regimen as induction therapy followed by 3 cycles of IDAC or HiDAC therapy. IDAC and HiDAC regimen were defined as i.v. Ara-C dose at 1000–2500 mg/m^2^/dose and 2501–3000 mg/m^2^/dose every 12 h for 3 days (d1, 3, 5), respectively. The dosage of Ara-C in patients receiving IDAC therapy was assigned to 1000 mg/m^2^/dose every 12 h on d1, 3, 5 for patients aged 60 years and older (elderly patients), whereas patients younger than 60 years who had previously experienced severe infection or unfit after the induction or the first cycle of consolidation therapy with 7 + 3 or 5 + 2 were treated with i.v. Ara-C 2000–2500 mg/m^2^/dose every 12 h on day 1, 3, 5. Conversely, fit patients aged < 60 years with or without t(8;21) received HiDAC regimen. Azacitidine 100 mg subcutaneous route for 7 days every 4 weeks were given as consolidation therapy until the disease relapse in elderly patients who were unfit for receiving IDAC therapy.

### Cytogenetic and molecular techniques

Short-term culture of BM cells, metaphase spread preparation and G-banding were performed. Karyotypes were described according to ISCN 2016 [7]. FLT3-ITD, NPM1, and CEBPA gene mutations were studied using genomic DNA isolated from BM or peripheral blood samples with QIAamp DNA Blood Mini Kit (Qiagen, Germany). FLT3 exon 14, 15 and 20 was amplified by PCR using specific primers and subsequently digested with EcoRV as previously described [8, 9]. The PCR products were analyzed by 2% agarose gel electrophoresis. NPM1 gene mutations were performed by fluorescent PCR [10], PCR products were separated by 3130 Genetic Analyzer (Applied Biosystem, USA), and the results were analyzed using Gene Mapper software version 4 (Applied Biosystems, USA). CEBPA gene mutations were investigated by amplification of entire coding region and bidirectional sequencing was performed using Bigdye Terminator Version 1.1 Cycle Sequencing Kit (Qiagen, Germany). Patient’s sequences were compared to CEBPA reference gene to evaluate the mutation status. The cytogenetic and molecular stratification risks were classified by 2017 ELN recommendations [2].

### Outcome assessment

The objectives of this study were to evaluate RFS and OS of the entire study population and subgroup analysis that we divided the study population into four subgroups according to the factors that would affect to RFS and OS, including AML patients receiving best supportive care, AML patients completed 4 cycles of consolidation therapy, cytogenetically normal AML patients with or without completed consolidation therapy. RFS was defined as the length of time from the date of CR to relapse. OS was defined as the interval between the dates of diagnosis and death.

### Statistical analysis

The parameters which included age, white blood cell (WBC) count, cytogenetics, molecular data and treatment regimens were compared between patients with and without CR by using Chi-square. OS and RFS were calculated using the Kaplan–Meier method, difference between groups were calculated using the log-rank test for univariate analysis. Cox’s Regression model was used for multivariate survival analysis. All calculations were performed using the statistical package of social sciences software, SPSS statistics version 17 (Chicago: SPSS Inc; 2008), p < 0.05 was considered significant.

## Results

### Cytogenetic and molecular analysis

A total of 141 AML patients, the median age at diagnosis was 54 years (15–88 years). Fifty-four patients (38%) were older than 60 years and 71 patients were male. Normal karyotype (NK), complex, favorable, intermediate and adverse chromosomal aberrations were found in 83 (59%), 23 (16%), 7 (5%), 19 (14%) and 9 patients (6%), respectively. Normal karyotype was identified in 64% (70 patients) and 74% (40 patients) of patients aged ≥ 40 years (109 patients) and patients aged ≥ 60 years (54 patients), respectively. Patients aged ≥ 40 years had significantly higher incidence of NK than those in patients aged < 40 years (*p* = 0.017). In group of patients aged < 40 years (32 patients), NK, favorable, intermediate and adverse cytogenetic abnormalities were observed in 13 (41%), 4 (12.5%), 5 (15.5%) and 10 patients (31%), respectively. In contrast, NK, favorable, intermediate and adverse risk cytogenetic abnormalities in patients aged ≥ 40 years were detected in 70 (64%), 3 (3%), 14 (13%) and 22 (20%) out of 109 patients. Patients aged < 60 years had more favorable (7% versus 2%) and adverse risk cytogenetics (30% versus 11%) than those in patients aged ≥ 60 years, whereas, elderly patients (aged ≥ 60 years) expressed more intermediate risk cytogenetics (87% versus 63%) with 75% of NK, *p* = 0.009. Favorable risk cytogenetic in our patients was seen only t(8;21).

In this study, three gene mutation tests (NPM1, FLT3-ITD and CEBPA) were performed in all AML patients, 88 patients (62%) had no mutation and 53 patients (38%) had at least one gene mutation. Single gene mutation was found in 43 patients (30%); mutation of NPM1 (18 patients; 13%), FLT3-ITD (11 patients; 8%) and CEPBA genes (14 patients; 10%). Combination of NPM1 and FLT3-ITD gene mutations was found in 7 out of 141 patients (5%), the coexisting mutation of CEBPA/FLT3-ITD/NPM genes was detected in 1 patient (0.7%) and the remaining 2 patients (1.3%) had either mutant FLT3-ITD or NPM1 gene with CEBPA gene mutations.

In group of cytogenetically normal AML (CN-AML) patients, 48 out of 83 patients (57.8%) exhibited gene mutations, while only 5 out of 58 AML patients with cytogenetic abnormalities (9%) had somatic gene mutation, *p* < 0.001. Mutation of NPM1, FLT3-ITD and CEBPA genes in CN-AML were shown in 27 (33%), 15 (18%) and 16 (19%) out of 83 patients, respectively. Single and double mutations of CEBPA gene were found in 10 and 6 patients, respectively.

All patients harboring NPM1 mutation had NK, conversely, FLT3-ITD mutation was observed in NK (15 patients), complex chromosome (2 patients), 11q13 (1 patient) and t(7;11) (1 patient). All AML patients with CEBPA mutation except 1 had NK. Patients who had NK were significantly found mutated NPM1 (*p* < 0.001) or CEBPA (*p* = 0.002), but not significantly seen with FLT3-ITD mutation (*p* = 0.113). Median age in AML patients with FLT3-ITD, NPM1 and CEBPA mutations were 56, 60 and 57 years, respectively. Comparison between patients younger and older than 40 years, cytogenetic abnormalities were frequently seen in patients younger than 40 years (59% versus 36%), *p* = 0.017, whereas somatic gene mutation (≥ 1 gene) was shown significantly in patients aged ≥ 50 years compared with that in patients younger than 50 years (45% versus 24%), *p* = 0.009. Twenty four percent of patients aged ≥ 40 years had NPM1 mutation compared with that in patients aged < 40 years (3%), *p* = 0.009. There was no significant correlation between age and patients with FLT3-ITD or CEBPA mutation.

Median white blood cell (WBC) count at diagnosis in the entire study was 23,100/μL (1016–444,450/μL), patients harboring NPM1 and FLT3-ITD mutations had 2.5- and 6.0-fold higher median WBC counts in peripheral blood than those with wild-type NPM1 (53,000 versus 21,180/μL) and FLT3-ITD, (122,678 versus 20,185/μL), respectively, whereas, the median WBC count was similar in CEBPA mutated and wild-type CEBPA patients (27,000 versus 22,900/μL).

### Treatment outcomes

A total of 141 adult AML patients, 111 patients (79%) were treated with chemotherapy or hypomethylating agent and the remaining 30 patients (21%) received only best supportive care. Of the 111 treated patients, 84 (76%), 20 (18%) and 7 patients (6%) were treated with 7 + 3, 5 + 2 and azacitidine, respectively. Except 9 patients (8%) who died within 3 weeks after start induction chemotherapy, the results of 102 evaluable treated patients revealed that 73 patients (72%) achieved CR and 60 patients (59%) got CR after first induction. Eighty patients receiving 7 + 3 and sixteen patients treated with 5 + 2 regimens achieved CR in 61 (76%) and 12 patients (75%), respectively. Twenty-two patients failed to achieve CR after first induction chemotherapy were treated with the second course of 7 + 3, 5 + 2 or etoposide combined with mitoxantrone based regimen. There was no patient achieved CR after azacitidine therapy, nevertheless, all patients treated with azacitidine had BM blast at diagnosis > 60%.

Four CR patients refused treatment, therefore, only 69 out of 73 CR patients (94.5%) received first consolidation therapy; 50 patients (68%) received 7 + 3 or 5 + 2 regimen, 14 patients (19%) were treated with either HiDAC (9 patients) or IDAC (5 patients), and the remaining 5 patients (7%) received azacitidine (4 patients) and etoposide combined with mitoxantrone (1 patient). Finally only 41 out of 73 CR patients (56%) had completed 4 cycles of consolidation therapy, the remaining 32 patients did not complete consolidation therapy because of early relapse (9 patients), dead (6 patients), severe co-morbidities (2 patients) and refusing chemotherapy (15 patients). Of 41 patients who had completed consolidation therapy, 25 (61%), 12 (29%) and 4 patients (10%) r**e**ceived IDAC, HiDAC and azacitidine therapy, respectively. Twenty patients aged < 60 years had completed consolidation therapy with IDAC (2000–2500 mg/m^2^/dose), the remaining 5 elderly patients received IDAC (1000 mg/m^2^/dose). Consolidation therapy with azacitidine in 4 patients were given until disease relapse. HiDAC was given in 12 patients, 2, 2, 3 and 5 patients had t(8;21), complex chromosome, other abnormal cytogenetics and normal karyotype, respectively. Seventeen patients receiving IDAC were normal cytogenesis, while 2 patients had t(8;21) and 6 patients had other cytogenetic abnormalities. In IDAC consolidation group (25 AML patients), 2, 8 and 2 patients had FLT3-ITD, NPM1 and CEBPA mutation, respectively and 13 patients had no gene mutation. HiDAC consolidation was given in 11 AML patients without gene mutation and 1 patient who had CEBPA mutation. Azacitidine was administered in 3 AML patients without mutation and 1 patient with CEBPA mutation. Patients aged < 60 years (*p* = 0.019) and patients receiving induction chemotherapy with 7 + 3 or 5 + 2 (*p* < 0.001) were associated with significantly greater CR rate than those in patients aged ≥ 60 years and patients treated with azacitidine. Patients’ characteristics and factors affecting CR are shown in Table 1.

**Table 1:** Patients’ characteristics and factors affecting CR in 102 evaluable treated AML patients

Factors	Number of patients	Complete remission N (%)	*p*
Age (years)			
< 60	79	61 (77)	0.019
≥ 60	23	12 (52)	
White blood cell count			
< 100,000/μL	20	14 (70)	0.862
> 100,000/μL	82	59 (72)	
Cytogenetic risk			
Favorable	7	6 (86)	0.051
Intermediate	71	54 (76)	
Adverse	24	13 (54)	
Gene mutation			
FLT3-ITD	8	5 (63)	0.453
NPM1	13	11 (85)	
FLT3-ITD and NPM1	6	5 (83)	
CEBPA	10	7 (70)	
No mutation	64	46 (72)	
Co-existence of mutant CEBPA with other gene mutation	1	1 (100)	
Single FLT3-ITD mutation			
Yes	8	5 (63)	0.554
No	94	68 (72)	
Single NPM1 mutation			
Yes	13	11 (85)	0.100
No	89	62 (70)	
Single CEBPA mutation			
Yes	10	7 (70)	0.198
No	92	65 (71)	
Induction chemotherapy			
7 + 3	80	61 (76)	0.000
5 + 2	16	12 (75)	
Azacitidine	6	0	

**Fig. 1:**
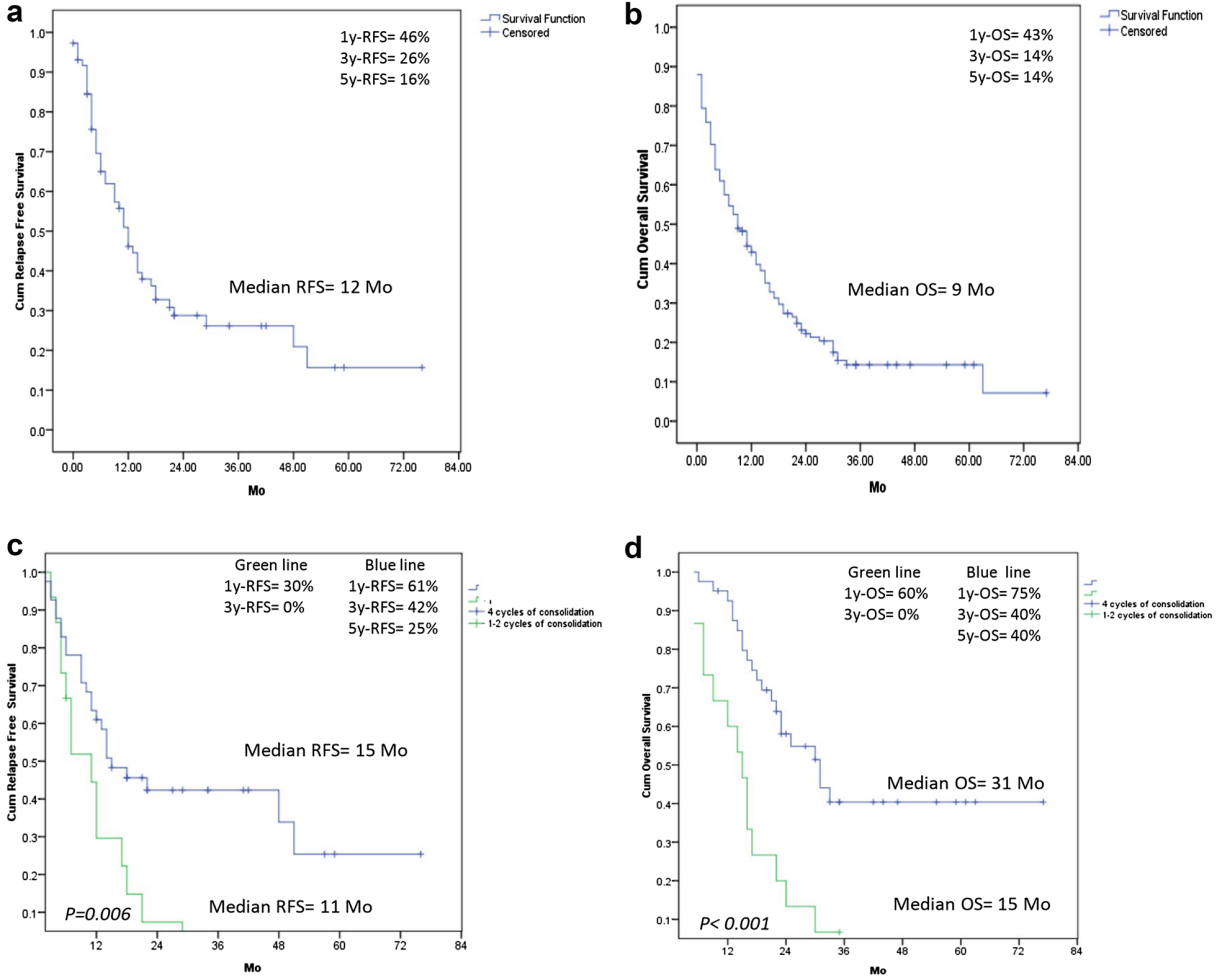
**a** Relapse free survival (RFS) in all 141 AML patients. **b** Overall survival (OS) in all 141 AML patients. **c** RFS in AML patients who were treated with 4 cycles and 1–2 cycles of consolidation chemotherapy (**d**) OS in AML patients who were treated with 4 cycles and 1–2 cycles of consolidation chemotherapy

**Fig. 2:**
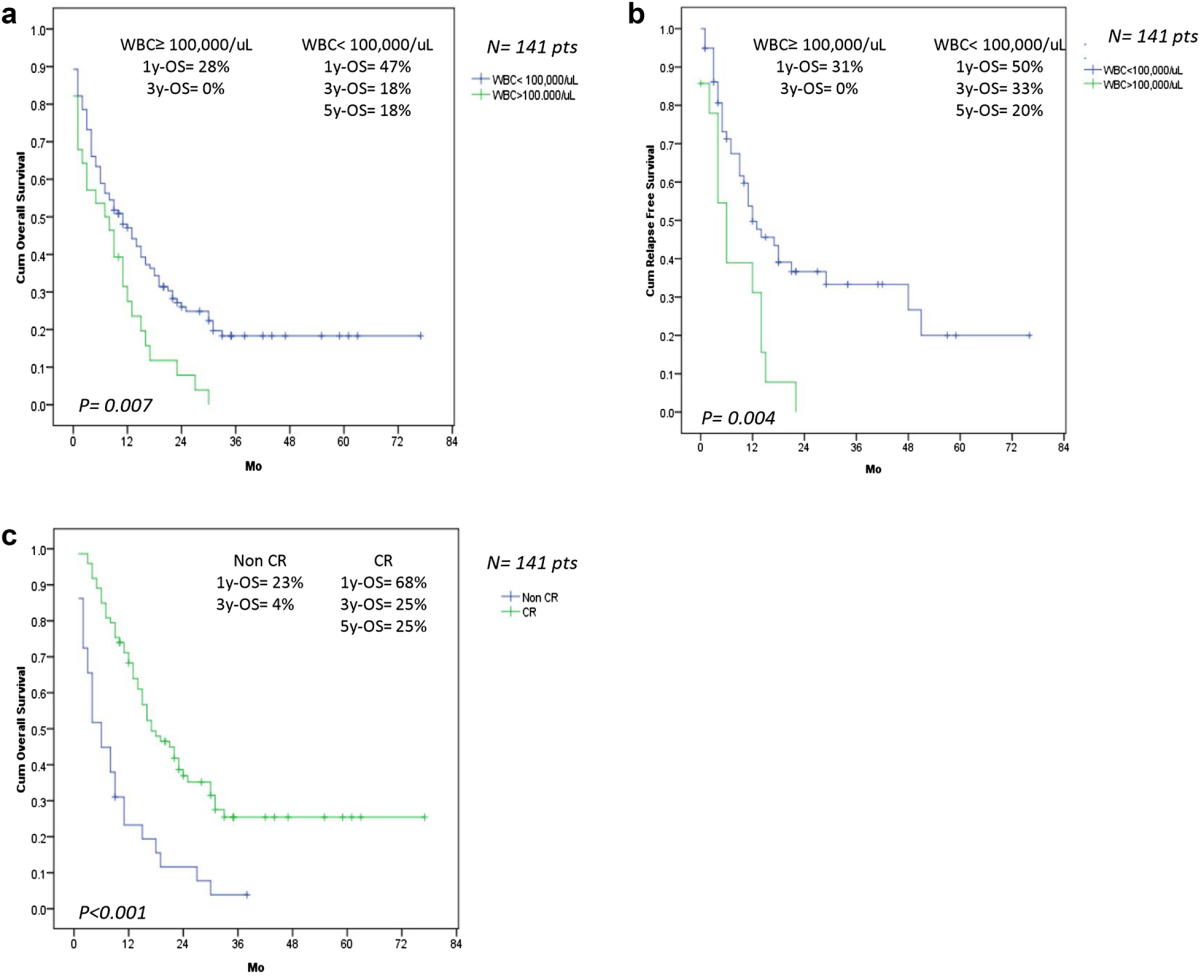
**a** OS in 141 AML patients who had WBC < 100,000/μL and WBC ≥ 100,000/μL. **b** RFS in 141 AML patients who had WBC < 100,000/μL and WBC ≥ 100,000/μL. **c** OS in 141 AML patients who achieved complete remission (CR) and non CR

A 5 year follow up, median RFS and OS were 12 and 9 months, respectively, and 5y-RFS was 16% and 5y-OS was 14% in the whole study population. Median OS in treated and untreated AML patients were 13 and 2 months, respectively. The median RFS and OS were significantly longer in patients treated with 4 cycles of consolidation chemotherapy (41 patients) than those treated with 1 or 2 cycles of consolidation therapy (17 patients); RFS were 15 and 11 months, respectively (*p* = 0.006) and OS were 31 and 15 months, respectively (*p* < 0.001). 5y-RFS and 5y-OS in group of patients completed consolidation therapy were 25 and 40%, respectively, which were significantly greater than those in the whole study population, *p* < 0.001 (Fig. 1). A total of 141 AML patients, WBC < 100,000/μL (*p* = 0.004) and wild-type FLT3-ITD (*p* = 0.047) were associated with significantly longer RFS than those in groups of WBC ≥ 100,000/μL and FLT3-ITD positive AML in the univariate analysis, however, they were not found to be independently significant in multivariate analysis. In univariate analysis of OS, five individual factors significantly affected OS in the overall study population in order of increasing significance, these included patients aged < 60 years, WBC < 100,000/μL, non adverse cytogenetic risk, wild type FLT3-ITD and CR patients, nevertheless, the multivariate analysis indicated only non adverse cytogenetic risk (*p* = 0.035) and CR patients (*p* < 0.001) were significantly favorable prognostic factors for OS. The status of high WBC count and CR affected the survival in 141 AML patients are shown in Fig. 2.

**Fig. 3:**
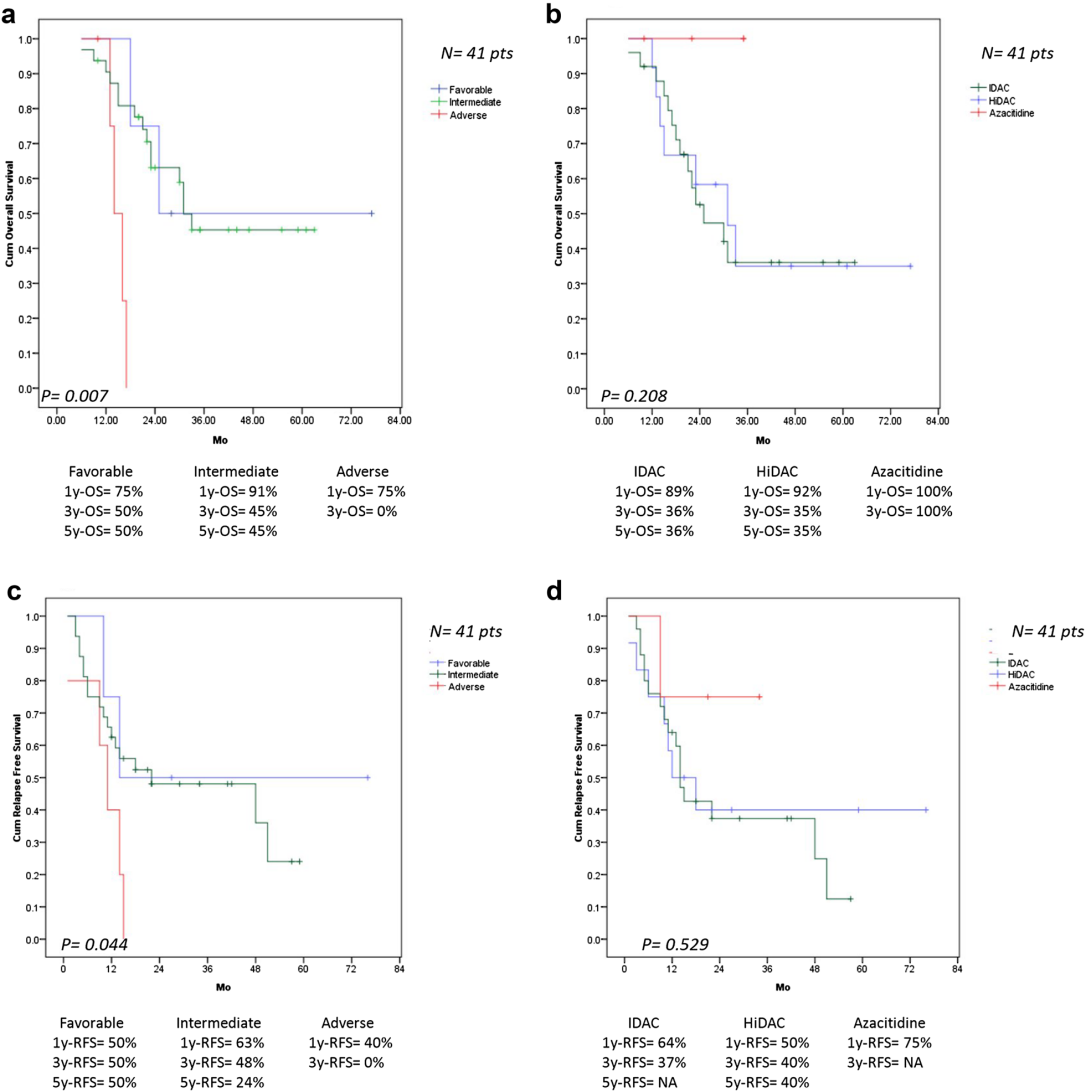
**a** OS in 41 AML patients with complete consolidation therapy classify by cytogenetic risk. **b** OS in 41 AML patients with complete consolidation therapy with HiDAC and IDAC regimen. **c** RFS in 41 AML patients with complete consolidation therapy classify by cytogenetic risk. **d** RFS in 41 AML patients with complete consolidation therapy with HiDAC and IDAC regimen

**Fig. 4:**
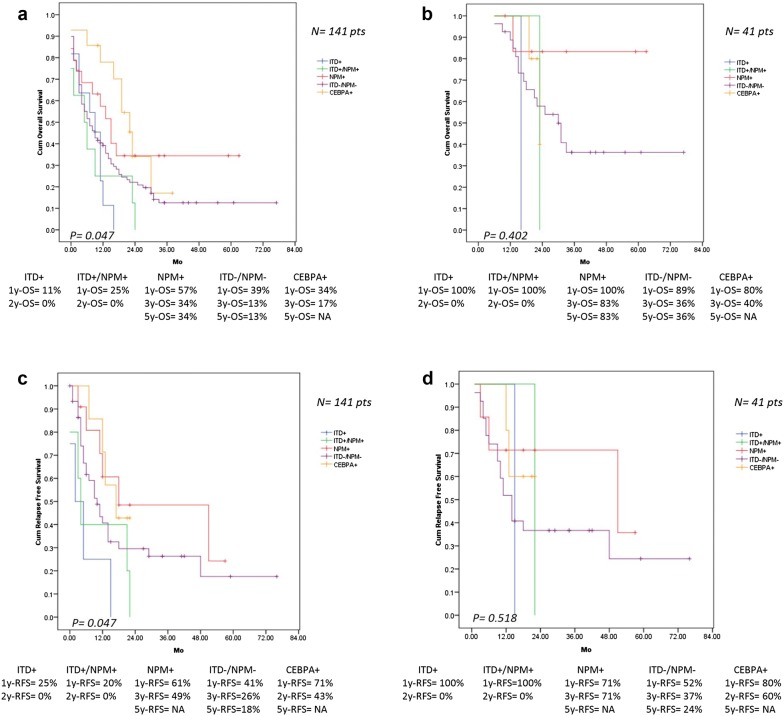
**a** OS in 141 AML patients classify by type of gene mutation. **b** OS in 41 AML patients with complete consolidation therapy classify by type of gene mutation. **c** RFS in 141 AML patients classify by type of gene mutation. **d** RFS in 41 AML patients with complete consolidation therapy classify by type of gene mutation

Among 41 AML patients who completed consolidation therapy, the results of univariate analysis showed that the patients with favorable and intermediate risk cytogenetics had prolonged RFS (*p* = 0.044) and OS (*p* = 0.007) than those with adverse karyotype. In CN-AML, consolidation with IDAC had longer median RFS than HiDAC, 22 versus 12 months. Nevertheless, consolidation with HiDAC had 5y-RFS than IDAC, 40% versus < 15% (Figs. 3, 4), and patients with WBC < 100,000/μL at diagnosis was also associated with prolonged OS (*p* = 0.013), but the difference was not significant in multivariate analysis.

A total of 83 patients with CN-AML, patients with WBC < 100,000/μL (*p* = 0.003) and wild-type FLT3-ITD (*p* < 0.001) were found to be significantly associated with increased RFS, while patients with WBC < 100,000/μL (*p* = 0.008) and patients aged < 60 years (*p* = 0.003) were associated with longer OS according to the univariate analysis. In multivariate analysis, shorter RFS was found in positive FLT3-ITD AML patients (*p* = 0.025) and shorter OS were observed in group of WBC ≥ 100,000/μL (*p* = 0.026) and non-CR patients (*p* = 0.027) (Table 2).

**Table 2:** Factors affecting relapse free and overall survival in 141 non-transplant AML patients

Factors	Overall study population				Completed consolidation therapy				AML with normal karyotype				AML with normal karyotype and completed consolidation therapy				Supportive care	
	mRFS (Mo) N = 73	*p*	mOS (Mo) N = 141	*p*	mRFS (Mo) N = 41	*p*	mOS (Mo) N = 41	*p*	mRFS (Mo) N = 42	*p*	mOS (Mo) N = 83	*p*	mRFS (Mo) N = 22	*p*	mOS (Mo) N = 23	*p*	mOS (Mo) N = 30	*p*
Age (years)																		
< 60	11	0.235	13	0.007	14	0.196	30	0.436	12	0.734	17	0.003	18	0.504	31	0.957	3	0.781
≥ 60	22		4		NR		NR		22		4		NR		NR		2	
White blood cell count																		
< 100,000/μL	12	0.004	11	0.007	48	0.074	33	0.013	18	0.003	15	0.008	48	0.029	63	0.017	2	0.12
> 100,000/μL	6		7		14		17		6		5		6		23		1	
Cytogenetic risk																		
Favorable	14	0.141	18	0.000	14	0.044	25	0.007	–	–	–	–	–	–	–	–	–	0.236
Intermediate	12		11		22		31										3	
Unfavorable	9		4		9		14										2	
Gene mutation																		
FLT3-ITD+	2	0.047	9	0.047	15	0.518	16	0.402	1	0.000	7	0.051	–	0.156	–	0.397	1	0.072
NPM1+	18		15		51		63		18		15		51		63		2	
FLT3-ITD+ and NPM1+	4		5		22		23		4		5		22		23		1	
CEBPA+	17		22		NR		23		17		22		NR		23		16	
No mutation	10		7		14		30		11		9		11		31		2	
FLT3-ITD mutation																		
Yes	5	0.04	7	0.076	15	0.686	16	0.131	1	0.000	7	0.074	–	–	–	–	1	0.416
No	12		9		18		31		13		13		22		31		2	
NPM1 mutation																		
Yes	18	0.16	15	0.178	51	0.146	63	0.222	18	0.115	15	0.27	51	0.061	63	0.161	1	0.665
No	11		9		14		30		12		11		14		31		2	
CEBPA mutation																		
Yes	13	0.439	19	0.149	NR	0.411	23	0.834	17	0.417	19	0.362	NR	0.601	23	0.547	16	0.016
No	11		9		14		31		12		11		22		33		2	
Consolidation regimen																		
HiDAC	12	0.257	31	0.535	1	0.529	31	0.208	12	0.188	31	0.273	12	0.203	31	0.281		–
IDAC	14		25		14		25		22		31		22		31			
Azacitidine	NR		NR		NR		NR		NR		NR		NR		NR			
Consolidation regimen																		
HiDAC	12	0.683	31	0.803	12	0.788	31	0.557	12	0.25	31	0.267	12	0.226	31	0.267		–
IDAC	14		25		14		25		22		31		22		31			
Complete remission (CR)																		
CR		–	17	0.000		–		–	–	–	23	0.005	–	–	–	–		–
No CR			6								9							

*mRFS* median relapse free survival, *mOS* median overall survival, *NR* not reach, *NA* not available

**Fig. 5:**
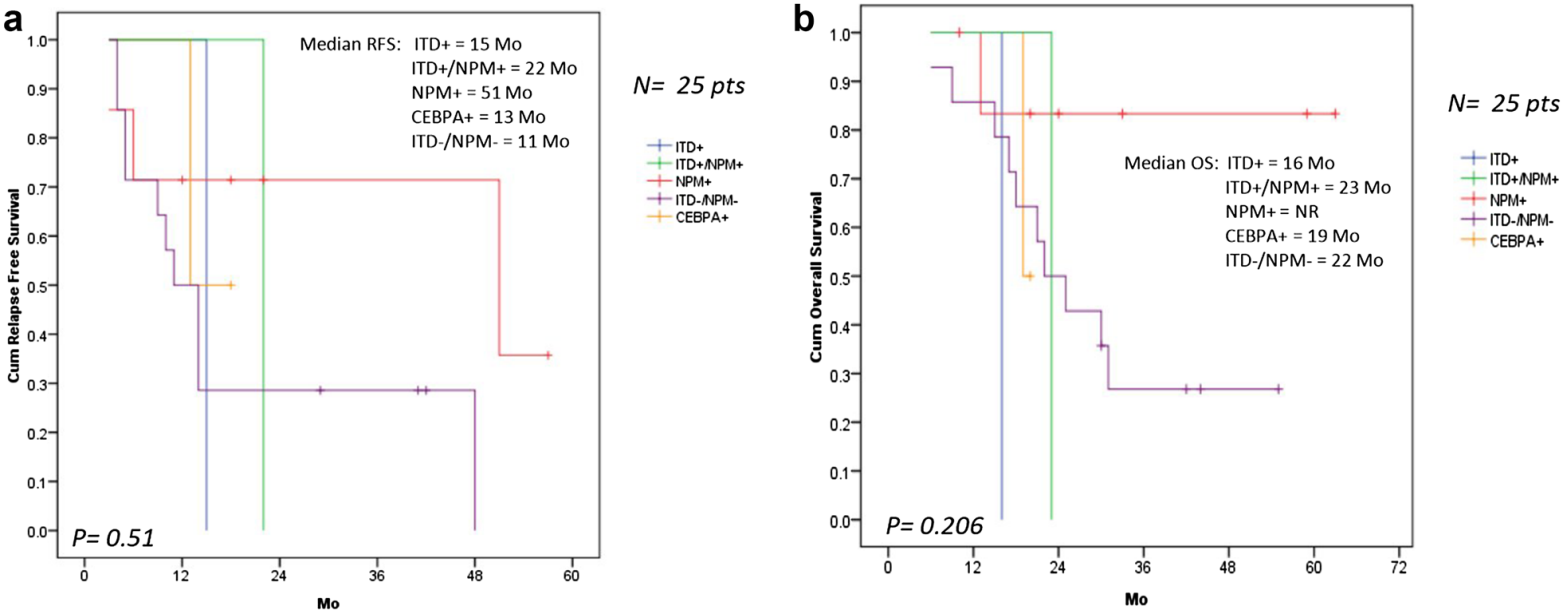
**a** RFS in AML patients receiving complete consolidation therapy with IDAC regimen classify by type of gene mutation. **b** OS in AML patients receiving complete consolidation therapy with IDAC regimen classify by type of gene mutation

In patients with CN-AML who completed consolidation therapy, only patients with WBC < 100,000/μL had longer RFS (*p* = 0.029) and OS (*p* = 0.017) than those with WBC ≥ 100,000/μL. Mutant NPM or CEBPA gene illustrated longer RFS and OS than those in wild type NPM or CEBPA, but the difference was not significant. In group of untreated patients (30 patient), CEBPA mutation was also a significant factor for prolonged OS (*p* = 0.016). Higher RFS and OS were found in biallelic CEBPA mutation compared to single CEBPA mutation in the entire study population and every subgroups of study but no statistically significant differences. The factors associated with survival of 141 AML patients are shown in Table 2. The impact of IDAC consolidation on survival in AML patients with and without gene mutations were analyzed and found that AML patients harboring FLT3-ITD and both FLT3-ITD and NPM1 mutations had poor RFS and OS compared with those in AML patients with mutant NPM1 or no mutated gene (Fig. 5). HiDAC was treated in 11 patients without mutation and only 1 patient with mutated CEBPA, therefore, we had no result on survival issue after HiDAC therapy in mutant NPM1 and FLT3-ITD AML (Table 3).

**Table 3:** Impacts of gene mutations on relapse free and overall survival in 37 AML patients receiving IDAC and HiDAC consolidation

Factors	Median RFS				1y-RFS		3y-RFS		5y-RFS		Median OS				1y-OS		3y-OS		5y-OS	
	IDAC (Mo)	*p*	HiDAC (Mo)	*p*	IDAC (%)	HiDAC (%)	IDAC (%)	HiDAC (%)	IDAC (%)	HiDAC (%)	IDAC (Mo)	*p*	HiDAC (Mo)	*p*	IDAC (%)	HiDAC (%)	IDAC (%)	HiDAC (%)	IDAC (%)	HiDAC (%)
Gene mutation																				
FLT3-ITD	15	0.51	–	0.640	100	–	0	–	0	–	16	0.206	–	0.982	100	–	0	–	0	–
NPM1	51		–		71	–	71	–	36	–	NR		–		100	–	83	–	83	–
FLT3-ITD and NPM1	22		–		100	–	0	–	0	–	23		–		100	–	0	–	0	–
CEBPA	13		12		100	50	NA	NA	NA	NA	19		23		100	100	NA	NA	NA	NA
No mutation	11		11		50	50	29	38	0	38	22		31		86	90	27	36	NA	36
FLT3-ITD mutation																				
Yes	15	0.813	–	–	100	–	0	–	0	–	16	0.192	–	–	100	–	0	–	0	–
No	14		12		61	50	42	40	14	40	30		31		91	92	40	35	40	35
NPM1 mutation																				
Yes	51	0.116	–	–	45	–	56	–	28	–	NR	0.116	–	–	100	–	69	–	69	–
No	14		12		59	50	29	40	0	40	22		31		88	92	25	35	25	35
CEBPA mutation																				
Yes	13	0.777	12	0.64	61	50	37	NA	NA	NA	19	0.797	23	0.982	100	100	NA	NA	NA	NA
No	14		11		50	50	NA	38	12	38	25		31		91	90	37	36	37	36

*NA* not available

## Discussion

This study demonstrated long-term outcomes of consolidation chemotherapy in adult AML patients who didn’t undergo transplantation, because of lack of an HLA matching donor, financial problem, unfit or older patients. The prognostic impact of cytogenetic abnormalities on survival have significant implication for AML even in the era of molecular risk stratification in AML [11–13]. Numerous somatic gene mutations have been reported as a potential tool to predict survival outcomes in cytogenetically normal AML [14–23]. Our results illustrated that normal cytogenetic was found in 59% of all de novo AML patients and it was observed more in patients aged ≥ 40 years. In group of patients aged < 40 years, 41% had cytogenetic abnormalities and found adverse cytogenetic abnormalities (31%) than intermediate (15.5%) and favorable risk (13%), despite almost all of them didn’t have AML with myelodysplasia related changes or prior history of myelodysplastic syndrome. The prevalence of CN-AML in this series was slightly higher than that in the other previous studies (42–48%) from MRC AML10 [11], CALGB 8461 [12, 15, 16], and in our previously published study which studied the treatment outcomes in 106 treated AML patients [24]. AML with adverse cytogenetic abnormalities had dismal RFS and OS, which were similar to those described in the previous reports from CALGB, US intergroup and MRC study groups [15].

CN-AML was significantly found in AML patients aged ≥ 40 years, and mutation of NPM1 and CEBPA genes were significantly exhibited in this group, therefore these driven gene mutations were associated with myeloid leukemia development in the middle aged and the older patients. High prevalence of CEBPA mutation was detected in our AML patients with 12% and 19% of the entire study population and CN-AML patients, respectively. On the contrary, mutated NPM1 and FLT3-ITD genes in the whole study population were shown in 19% (33% in CN-AML) and 14% (18% in CN-AML), which lower than those in the previous studies [1, 15, 25–28]. Nevertheless, the prevalence of NPM1, FLT3-ITD and CEBPA mutations in our patients were quite similar to those in Taiwanese and Chinese patients with de novo AML [5, 6, 28].

Patients with NPM1 or CEBPA mutation had longer RFS and OS compared with those in wild type NPM1 and CEBPA which were shown in the entire study population and in all subgroups of the study, these results were similar to the survival analysis in the previous studies [1, 14–16, 25]. CEBPA mutation was found positive impact on survival even in patients receiving supportive care therapy. Nevertheless, the overall survival in the whole study population with mutant NPM1 or CEBPA were lower than those in patients with completion of 4 cycles of consolidation chemotherapy, and in the previous studies [6, 14–16, 29], which the causes of shorter OS in the entire population was numerous patients died from febrile neutropenia with bacterial sepsis and few patients also refused chemotherapy. FLT3-ITD mutant AML was significantly associated with shorter RFS in the whole study population (5 months) and in a group of CN-AML (1 month), however, the different of RFS between FLT3-ITD mutation (15 months) and wild type FLT3-ITD (18 months) was not significant in the group of patients with completed consolidation therapy, that might be from small number of our AML patients with mutant FLT3-ITD who had completed consolidation chemotherapy. Comparison between FLT3-ITD mutant AML and wild-type FLT3-ITD, shorter OS was seen in AML patients with mutated FLT3-ITD in all subgroups analysis but no statistically significant difference. Twenty-eight percent of all AML treated patients had primary chemotherapy resistance and only one-third of AML treated patients (56% of CR patients) had completed 4 cycles of consolidation chemotherapy.

In non-transplant AML patients, patients receiving 4 cycles of consolidation therapy had longer RFS and OS than those treated with 1 or 2 cycles of consolidation chemotherapy. In the whole study population, patients receiving HiDAC consolidation tended to have better long term RFS than that in patients treated with IDAC, but there was no difference in OS between these two groups (Figs. 3, 4). These results were congruous with the previous reports [30–32]. Interestingly, patients receiving induction chemotherapy followed by azacitidine consolidation appeared to have longer RFS and OS than consolidation with chemotherapy, but there was no statistically significant difference because of short follow up duration of azacitidine group, small number of patients receiving azacitidine and patients completed consolidation chemotherapy.

Elderly patients had longer OS than younger patients, this may be because most elderly patients had normal cytogenetic (75%) and had low incidence of adverse cytogenetics (11%). Besides, they received consolidation with IDAC or azacitidine, which were low intensity, less myelosuppressive and severe infection complications. Thus azacitidine might be suitable for consolidation therapy in older AML patients. WBC ≥ 100,000/μL was also significantly related to shorter RFS and OS in the whole study population and all subgroup analysis, however, the number of WBC did not affect the CR rate and patients achieving CR had longer OS compared with that in patients who failed to achieve CR.

The limitation of this study were a retrospective study, small number of patients who completed 4 cycles of consolidation therapy and short follow up duration in AML patients with CEBPA mutation, however, the consolidation therapy regimen in this study was chosen follow the patients’ status during AML treatment without selection bias which representing the real results in the clinical practice under limited resource.

## Conclusions

Cytogenetic and mutation test for FLT3-ITD, NPM1 and CEBPA genes were useful for identify prognostic outcomes in adult AML. Adverse cytogenetic abnormalities and FLT3-ITD mutation exhibited dismal RFS and OS.

## Abbreviations

FLT3-ITD: FMS related tyrosine kinase 3-internal tandem duplications
NPM1: nucleophosmin1
CEBPA: CCAAT/enhancer binding protein α gene
HiDAC: high dose Ara-C
IDAC: intermediate dose Ara-C

## References

[1] Schlenk RF, Döhner K, Krauter J, Fröhling S, Corbacioglu A, Bullinger L (2008). Mutations and treatment outcome in cytogenetically normal acute myeloid leukemia. N Engl J Med.

[2] Döhner H, Estey E, Grimwade D, Amadori S, Appelbaum FR, Büchner T (2017). Diagnosis and management of AML in adults: 2017 ELN recommendations from an international expert panel. Blood.

[3] Ling Y, Xie Q, Zhang Z, Zhang H (2018). Protein kinase inhibitors for acute leukemia. Biomark Res.

[4] Wu M, Li C, Zhu X (2018). FLT3 inhibitors in acute myeloid leukemia. J Hematol Oncol.

[5] Gu R, Yang X, Wei H (2018). Molecular landscape and targeted therapy of acute myeloid leukemia. Biomark Res.

[6] Wei H, Wang Y, Zhou C, Lin D, Liu B, Liu K (2018). Distinct genetic alteration profiles of acute myeloid leukemia between Caucasian and Eastern Asian population. J Hematol Oncol.

[7] McGowan-Jordan J, Simons A, Schmid M (2016). ISCN 2016: an international system for human cytogenetic nomenclature.

[8] Kiyoi H, Naoe T, Yokota S, Nakao M, Minami S, Kuriyama K (1997). Internal tandem duplication of FLT3 associated with leukocytosis in acute promyelocytic leukemia. Leukemia.

[9] Ali A, Siddique MK, Shakoori AR (2013). Frequency of FLT3/ITD mutations in pakistani acute myeloid leukemia patients. Pak J Zool.

[10] Machado-Neto JA, Traina F, Lazarini M, Melo Campos PD, Barbosa Pagnano KB, Lorand-Metze I (2011). Screening for hotspot mutations in PI3K, JAK2, FLT3 and NPM1 in patients with myelodysplastic syndromes. Clinics.

[11] Grimwade D, Walker H, Oliver F, Wheatley K, Harrison C, Harrison G, on behalf of the Medical Research Council Adult and Children’s Leukaemia Working Parties (1998). The importance of diagnostic cytogenetics on outcome in AML: analysis of 1612 patients entered into the MRC AML10 trial. Blood.

[12] Byrd JC, Mrozek K, Dodge RK, Carroll AJ, Edwards CG, Arthur DC (2002). Pretreatment cytogenetic abnormalities are predictive of induction success, cumulative incidence of relapse, and overall survival in adult patients with de novo acute myeloid leukemia: results from cancer and leukemia group B (CALGB 8461). Blood.

[13] Grimwade D, Hills RK, Moorman AV, Walker H, Chatters S, Goldstone AH (2010). Refinement of cytogenetic classification in acute myeloid leukemia: determination of prognostic significance of rare recurring chromosomal abnormalities among 5876 younger adult patients treated in the United Kingdom medical research council trials. Blood.

[14] Patel JP, Gonen M, Figueroa ME, Fernandez H, Sun Z, Racevskis J (2012). Prognostic relevance of integrated genetic profiling in acute myeloid leukemia. N Engl J Med.

[15] Ofran Y, Rowe JM (2013). Genetic profiling in acute myeloid leukaemia—where are we and what is its role in patient management. Br J Haematol.

[16] Hou HA, Lin CC, Chou WC, Liu CY, Chen CY, Tang JL (2014). Integration of cytogenetic and molecular alterations in risk stratification of 318 patients with de novo non-M3 acute myeloid leukemia. Leukemia.

[17] Liu WJ, Tan XH, Luo XP, Guo BP, Wei ZJ, Ke Q (2014). Prognostic significance of Tet methylcytosine dioxygenase 2 (TET2) gene mutations in adult patients with acute myeloid leukemia: a meta-analysis. Leuk Lymphoma.

[18] Bowen DT, Frew ME, Hills R, Gale RE, Wheatley K, Groves MJ (2005). RAS mutation in acute myeloid leukemia is associated with distinct cytogenetic subgroups but does not influence outcome in patients younger than 60 years. Blood.

[19] Marcucci G, Maharry K, Wu YZ, Radmacher MD, Mrózek K, Margeson D (2010). IDH1 and IDH2 gene mutations identify novel molecular subsets within de novo cytogenetically normal acute myeloid leukemia: a cancer and leukemia group B study. J Clin Oncol.

[20] Schnittger S, Dicker F, Kern W, Wendland N, Sundermann J, Alpermann T (2011). RUNX1 mutations are frequent in de novo AML with noncomplex karyotype and confer an unfavorable prognosis. Blood.

[21] Tang JL, Hou HA, Chen CY, Liu CY, Chou WC, Tseng MH (2009). AML1/RUNX1 mutations in 470 adult patients with de novo acute myeloid leukemia: prognostic implication and interaction with other gene alterations. Blood.

[22] Mendler JH, Maharry K, Radmacher MD, Mrózek K, Becker H, Metzeler KH (2012). RUNX1 mutations are associated with poor outcome in younger and older patients with cytogenetically normal acute myeloid leukemia and with distinct gene and MicroRNA expression signatures. J Clin Oncol.

[23] Gaidzik VI, Bullinger L, Schlenk RF, Weber D, Paschka P, Hahn J (2011). RUNX1 mutations in acute myeloid leukemia: results from a comprehensive genetic and clinical analysis from the AML study group. J Clin Oncol.

[24] Niparuck P, Chuncharunee S, Ungkanont A, Udomtrupayakul U, Aungchaisuksiri P, Rerkamnuatchoke B (2009). Long-term outcomes of de novo acute myeloid leukemia in Thai patients. J Med Assoc Thai.

[25] Mrezek K, Marcucci G, Paschka P, Whitman SP, Bloomfield CD (2007). Clinical relevance of mutations and gene-expression changes in adult acute myeloid leukemia with normal cytogenetics: are we ready for a prognostically prioritized molecular classification?. Blood.

[26] Abu-Duhier M, Goodeve AC, Wilson GA, Care RS, Peake IR, Reilly JT (2001). Genomic structure of human FLT3: implications for mutational analysis. Br J Haematol.

[27] FrThling S, Schlenk RF, Breitruck J, Benner A, Kreitmeier S, Tobis K (2002). Prognostic significance of activating FLT3 mutations in younger adults (16 to 60 years) with acute myeloid leukemia and normal cytogenetics: a study of the AML study group Ulm. Blood.

[28] Lin PH, Li HY, Fan SC, Yuan TH, Chen M, Hsu YH (2017). A targeted next-generation sequencing in the molecular risk stratification of adult acute myeloid leukemia: implications for clinical practice. Cancer Med.

[29] Ostronoff F, Othus M, Lazenby M, Estey E, Appelbaum FR, Evans A (2015). Prognostic significance of NPM1 mutations in the absence of FLT3-internal tandem duplication in older patients with acute myeloid leukemia: a SWOG and UK national cancer research institute/medical research council report. J Clin Oncol.

[30] Burnett AK, Russell NH, Hills RK, Hunter AE, Kjeldsen L, Yin J (2013). Optimization of chemotherapy for younger patients with acute myeloid leukemia: results of the medical research council AML15 trial. J Clin Oncol.

[31] Lowenberg B (2013). Sense and nonsense of high-dose cytarabine for acute myeloid leukemia. Blood.

[32] Schaich M, Rollig C, Soucek S, Kramer M, Thiede C, Mohr B (2011). Cytarabine dose of 36 g/m^2^ compared with 12 g/m^2^ within first consolidation in acute myeloid leukemia: results of patients enrolled onto the prospective randomized AML96 study. J Clin Oncol.

